# Liver Abscess Complicated with an Inflammatory Myofibroblastic Tumor

**DOI:** 10.5334/jbsr.2756

**Published:** 2022-09-20

**Authors:** Susana Rodrigues, Fábio Ferreira, Sílvia Dias

**Affiliations:** 1 Instituto Português de Oncologia Francisco Gentil Porto, PT; 2 Centro Hospitalar Universitário de São João, Porto, PT

**Keywords:** liver, inflammatory myofibroblastic tumors, hepatic abscess, paediatric

## Abstract

Inflammatory myofibroblastic tumors have a wide spectrum of biological behaviour and are composed of inflammatory cells and myofibroblastic spindle cells. Tumors may infrequently involve the liver. Imaging findings are non-specific.

**Teaching Point:** Radiologists should be familiar with inflammatory myofibroblastic tumors as a diagnostic consideration to avoid unnecessary surgery.

## Introduction

Inflammatory myofibroblastic tumor (IMT) of the liver is a rare and benign lesion with an unknown pathogenesis and etiology, but in some cases IMT is thought to result from an inflammation response to an underlying infection [1]. Actinomycetes were found to be associated with IMT in the liver, but there have been case reports of IMT associated with infections caused by other organisms. Inflammation resulting from trauma or surgery or in association with other malignancy are other possible etiologies [2].

It is difficult to obtain a diagnosis only by imaging, and radiological features may suggest malignant neoplasms. So histological examination is mandatory for a definitive diagnosis.

## Case History

**Figure 1:**
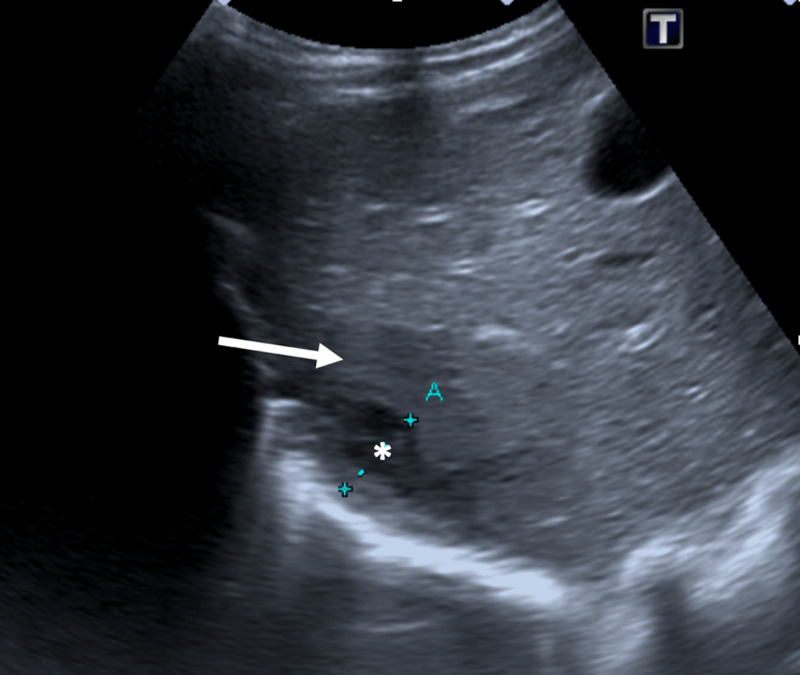
Initial evaluation with abdominal ultrasound showed a complex lesion with an hypoechogenic ill-defined area (arrow) and a more peripheral cystic component (*) in the right liver lobe.

**Figure 2:**
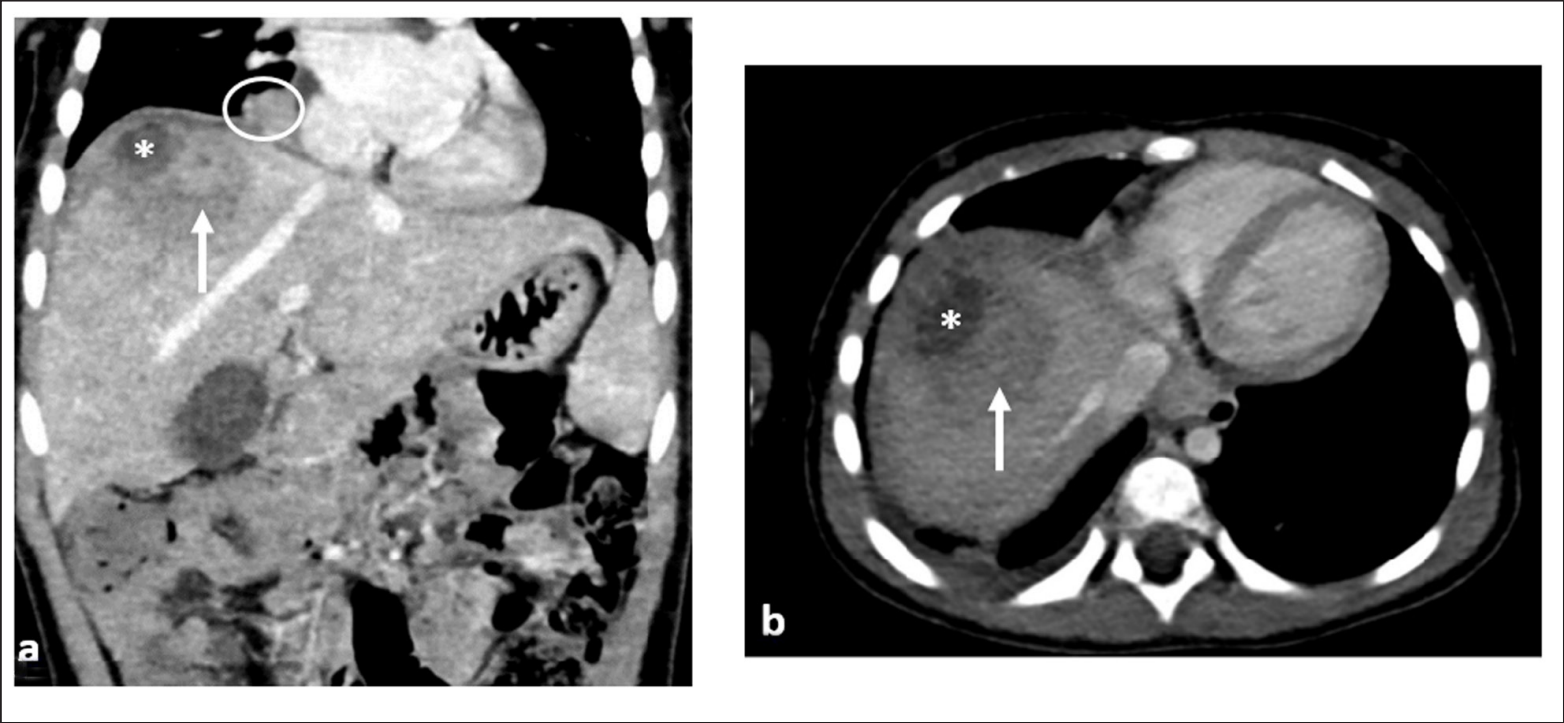
Enhanced coronal **(a)** and axial **(b)** CT show a more peripheral sub-diaphragmatic non-enhancing hypodense lesion in the right lobe, with extra-hepatic extension (*) and a more central enhancing solid mass (arrow). Presence of peri-hepatic ascites and cardiophrenic adenomegaly (circle).

A 3-year-old girl presented at the urgency department with persistent fever. The medical and surgical history was unremarkable. C-reactive protein levels and white cell count were elevated. Alpha fetoprotein was within the normal ranges. At initial evaluation, an abdominal ultrasound showed a hypoechogenic tumor and a peripheral cystic complex lesion in the right liver lobe (Figure 1). The hypothesis of hepatic abscess was considered. Computed tomography (CT) showed a contrast-enhancing lesion centred on the right hepatic lobe associated to an area of liquefaction and a subdiaphragmatic lenticular cystic component with extrahepatic extension. Multiple mediastinal adenomegaly and peri-hepatic effusion were presented (Figure 2).

**Figure 3:**
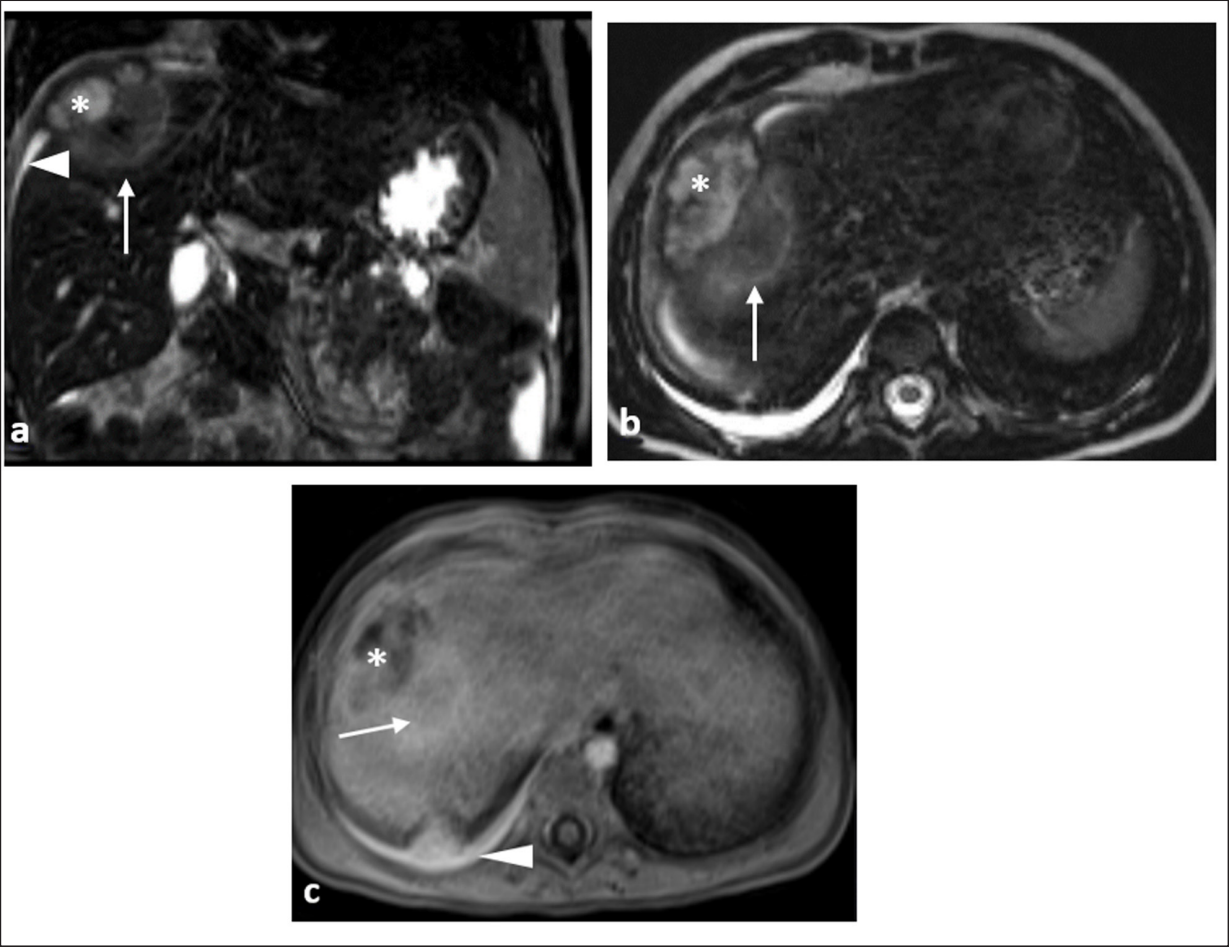
Coronal **(a)** and axial **(b)** 3D TSE sequence MR images show a mild hyperintense mass centred in right lobe, which shows progressively rim-like peripheral and central enhancement (arrows) at axial gadolinium-enhanced T1-weighted fat-saturated **(c)**. Periferically, a more cystic component (*), with enhancing walls and a non-enhancing center, compatible with an hepatic abcess. Right pleural and peri-hepatic effusions are also seen (arrowhead).

For further evaluation, abdominal magnetic resonance imaging (MRI) of the liver was performed 14 days later, which demonstrated an enhancing mass on segment VIII and a wall enhancing complex cystic lesion with extra-hepatic extension (Figure 3).

**Figure 4:**
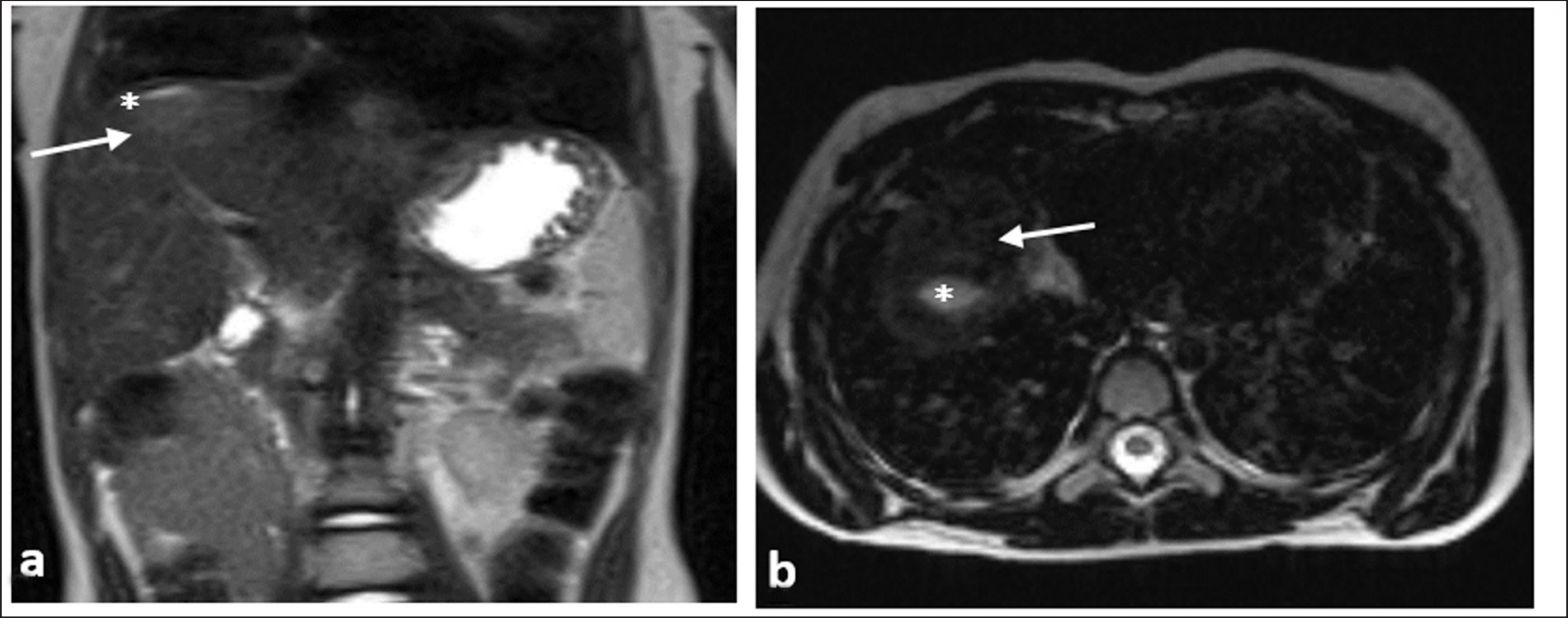
Coronal **(a)** T2-weighted fat-saturated single shot turbo spin-echo MR image and axial **(b)** 3D TSE sequence show regression of both hiperinytense cystic (*), and mild hiperintense solid components (arrows), after 6 weeks flucloxacillin therapy.

A CT-guided drainage of the cystic component and a histologic biopsy of the solid component were performed. The microbiological study of aspirated liquid from the cystic component revealed a methicillin-susceptible Staphylococcus Aureus. The histopathological and immunohistochemical findings were compatible with inflammatory myofibroblastic tumor with expression of caldesmon and actin. This patient was diagnosed with a liver abscess complicated by the presence of an inflammatory myofibroblastic tumor. The antibiotherapy were optimized and it was decided at the multidisciplinary group consultation to re-evaluate with MRI after the cessation of antibiotic therapy. Five months after the initial diagnosis, MRI revealed a significant dimensional regression of both intra and extra-hepatic components of the lesion (Figure 4), with a favourable outcome.

## Comment

Inflammatory myofibroblastic tumors are uncommon in liver and occur more frequently in children and young adults and affect males more than females [3]. It is difficult to distinguish them clinically and radiologically from malignant liver tumors [3,4]. The important entities included in the differential diagnosis are hepatocellular carcinoma and hepatoblastoma. Local recurrence and metastases may occur [5].

IMTs may be associated with fever, abdominal pain or a palpable mass [5]. The imaging findings are non-specific and they are often misdiagnosed as malignant neoplasms. Serum alphafetoprotein can help to differentiate IMT from hepatoblastoma or hepatocellular carcinoma. CT and MRI are the most used imaging tools in the evaluation of IMTs. This entity can present as a single or multifocal mass, being the solitary solid tumors the most common presentation and generally arises from the right liver [5]. Histological biopsy is imperious for a definitive diagnosis, and it can be seen spindle cells, which is the key feature, associated with a variable infiltrate of inflammatory cells and fibrous tissue [2,6].

If the biopsy proves the diagnosis of IMTs and it excludes malignancy, observation, and medical treatment with no steroidal anti-inflammatory drugs and antibiotics may be prescribed in patients with peripheral liver IMTs [6].

## Conclusion

The knowledge of this entity is fundamental because it can affect treatment. If radiological and histologic findings are compatible with IMT, the patients can be treated with observation in an initial phase. Surgical resection is indicated if the lesion persists or progresses after a conservative therapy or manifest malignant transformation. Complete resection is curative in most patients [6].

## References

[1] Dehner LP. The enigmatic inflammatory pseudotumors: The current state of understanding, or misunderstanding (editorial). J Pathol. 2000; 192: 277–279. DOI: 10.1002/1096-9896(200011)192:3<277::AID-PATH749>3.0.CO;2-E 11054708

[2] Narla LD, Newman B, Spottswood SS, Narla S, Kolli R. Infammatory pseudotumor. RadioGraphics. 2003; 23(3): 719–729. DOI: 10.1148/rg.233025073 12740472

[3] Durmus T, Kamphues C, Blaeker H, et al. Inflammatory myofibroblastic tumor of the liver mimicking an infiltrative malignancy in computed tomography and magnetic resonance imaging with Gd-EOB. Acta Radiol Short Rep. 2014; 3(7): 2047981614544404. DOI: 10.1177/2047981614544404 PMC4184414 25298878

[4] Yu J, Park C, Kim J, et al. Inflammatory myofibroblastic tumors in the liver: MRI of two immunohistochemically verified cases. J Magn Reson Imaging. 2007; 26: 418–421. DOI: 10.1002/jmri.21023 17623877

[5] Kauczor HU, Parizel P, Peh W. Imaging of the Liver and Intra-hepatic Biliary Tract – Volume 2: Tumoral Pathologies. Editor Emilio Quaia, 2021. ISSN 0942-5373. DOI: 10.1007/978-3-030-39021-1

[6] Cantera J, Alfaro M, Rafart D, et al. Inflammatory myofibroblastic tumours: A pictorial review. Insights Imaging. 2015; 6: 85–96. DOI: 10.1007/s13244-014-0370-0 25519466 PMC4330239

